# Digital Phenotyping for Real-Time Monitoring of Nonsuicidal Self-Injury Thoughts

**DOI:** 10.1192/j.eurpsy.2025.1971

**Published:** 2025-08-26

**Authors:** C.-Y. Ahn, J.-S. Lee

**Affiliations:** 1 Department of Psychology, Kangwon National University, Chuncheon-si, Korea, Republic Of

## Abstract

**Introduction:**

Digital phenotyping offers a valuable method for predicting and preventing nonsuicidal self-injury (NSSI) in daily life by providing objective, ecologically valid measurements at multiple time points. This approach quantifies an individual’s phenotype by capturing self-injury-related markers such as mood, step counts, and heart rate.

**Objectives:**

The aim of this study is to identify real-time predictors and to elucidate the dynamic trajectory of NSSI in individuals.

**Methods:**

This study targets individuals in their 20s residing in South Korea who engaged NSSI on five or more days during the past year, and a total of 56 participants were included in the current study. Once participants were enrolled, active (e.g., ecological momentary assessment) and passive (e.g., heart rate, step count) data were collected via a smartphone app and wrist-worn wearables for 14 days. Initially, a random forest algorithm was employed to assess the relative importance of passive and active data in predicting NSSI thoughts. Subsequently, a multilevel logistic regression model was used to capture variability at both the within-person and between-person levels.

**Results:**

After inputting passive data into the random forest algorithm, the model demonstrated an accuracy of 0.619. Among the variables, walking stride had the highest importance score at 0.28, followed by heart rate (0.18) and heart rate variability (0.17). Subsequently, when analyzing the random forest algorithm with active variables, the model’s accuracy was found to be 0.666. In this case, anger toward others had the highest importance score at 0.26, followed by depression (0.22) and anger toward oneself (0.19). In a separate analysis using multilevel logistic regression models for each passive variable, none of the variables produced significant results in either the fixed or random effects analyses. However, when active variables were entered into separate multilevel logistic regression models, all emotional variables yielded significant results in the fixed effects analysis: depression (0.746, p < .001), anxiety (0.521, p < .001), anger toward oneself (0.475, p < .001), anger toward others (0.403, p < .001), loneliness (0.329, p < .001), and shame (0.557, p < .05). In contrast, none of the variables showed significant results in the random effects analysis.

**Conclusions:**

The findings from this study could offer insights into novel mechanisms underlying the occurrence of self-injurious thoughts and their prediction in daily life. Additionally, this advanced approach may help identify optimal strategies for NSSI prevention and enable the delivery of personalized, real-time interventions.

**Disclosure of Interest:**

None Declared

